# SFRP2 suppresses trophoblast cell migration by inhibiting the Wnt/β‑catenin pathway

**DOI:** 10.3892/mmr.2024.13190

**Published:** 2024-02-28

**Authors:** Ruihong Lan, Yihong Yu, Jie Song, Mengdi Xue, Humin Gong

**Affiliations:** 1 Department of Obstetrics, Hainan General Hospital, Hainan Affiliated Hospital of Hainan Medical University, Haikou, Hainan 570311, P.R. China; 2 School of Clinical Medicine, Hainan Medical University, Haikou, Hainan 571199, P.R. China

**Keywords:** preeclampsia, secreted frizzled-related protein 2, migration, Wnt

## Abstract

The present study investigates the role of Secreted Frizzled-Related Protein 2 (SFRP2) in trophoblast cells, a key factor in preeclampsia (PE) progression. Elevated levels of Secreted Frizzled-Related Protein 1/3/4/5 (SFRP1/3/4/5) are associated with PE, but the role of SFRP2 is unclear. We analyzed SFRP2 expression in PE placental tissue using the GSE10588 dataset and overexpressed SFRP2 in JEG-3 cells via lentiviral transfection. The viability, migration, apoptosis, and proliferation of SFRP2-overexpressing JEG-3 cells were assessed using Cell Counting Kit-8, Transwell assays, flow cytometry, and EdU staining. Additionally, we evaluated the impact of SFRP2 overexpression on key proteins in the Wnt/β-catenin pathway and apoptosis markers (Bax, cleaved-caspase 3, BCL-2, MMP9, E-cadherin, Wnt3a, Axin2, CyclinD1, c-Myc, p-β-catenin, β-catenin, phosphorylated Glycogen Synthase Kinase 3 beta (p-GSK3β), and GSK3β) through western blotting. Results showed high SFRP2 mRNA and protein expression in PE placenta and JEG-3 cells post-transfection. SFRP2 overexpression significantly reduced JEG-3 cell viability, proliferation, and migration, while increasing apoptosis. It also altered expression levels of Wnt pathway proteins, suggesting SFRP2′s potential as a therapeutic target for PE by inhibiting trophoblast cell migration through the Wnt/β-catenin signaling cascade.

## Introduction

Preeclampsia (PE) is a cryptogenic multisystem disorder affecting 2–5% of the global gravid population and is characterized predominantly by hypertension and proteinuria emerging subsequent to the 20th week of gestation (1). PE is the third highest causative factor for maternal and neonatal morbidity and mortality, accounting for >60,000 maternal fatalities annually on a global scale (2,3). Presently, there is a lack of effective therapeutic or preventative treatment for PE, primarily due to its poorly understood pathogenesis (4,5). Key pathophysiological features of PE include increased apoptosis of trophoblast cells and reduced trophoblast invasion, leading to inadequate remodeling of spiral arteries (6,7).

Trophoblast cells form the fetal portion of the placental architecture (8). Dysregulation of these cells can result in adverse pregnancy outcomes. Specifically, decreased proliferation, excessive apoptosis and inadequate invasion of trophoblasts are associated with PE pathogenesis (9,10). Given the critical role of trophoblasts in embryo development, genes modulating the behavior of trophoblast cells are key in PE progression (11,12).

The Wnt/β-catenin signaling cascade is a key pathway in cellular processes, including cell proliferation, differentiation, migration, survival and apoptosis (13). Activation of Wnt/β-catenin pathways enhances trophoblast invasiveness (14). Secreted frizzled-related proteins (SFRPs), a family of five distinct members (SFRP1-5), are extracellular modulators known for inhibition of the Wnt signaling pathway (15). SFRPs influence various cellular processes by attenuating Wnt/β-catenin pathway activity (16). For example, SFRP4 inhibits aggressive traits of hepatocellular carcinoma (HCC) cells by reducing β-catenin levels, thereby blocking the Wnt/β-catenin signaling pathway (17). The reduced expression of sFRP-2 contributes to the advancement of esophageal basaloid squamous cell carcinoma by activating the β-catenin/Wnt signaling pathway (18). Increased expression of SFRP1 and SFRP3 proteins has been observed in placentas with intrauterine growth restriction (19), suggesting their involvement in the pathophysiology of placental disorder (19). Moreover, elevated transcriptional activity of SFRP4 in the placenta is closely linked with the etiology of severe PE, potentially due to its inhibitory interaction with Wnt2 (20). Additionally, increased expression of SFRP5 correlates with reduced invasiveness of trophoblast cells (21).

SFRP2, located on chromosome 4 (4q31.3) and composed of three exons and two introns, is a potent antagonist of the Wnt signaling cascade (22). SFRP2 has been associated with the initiation and progression of various cancers, including non-small cell lung cancer cells, colorectal cancer and glioma, primarily through its inhibition of the Wnt pathway (23–25). However, the functional role of SFRP2 gene in trophoblast cells during PE progression remains unclear. The present study aimed to investigate the role and mechanism of SFRP2 in trophoblast cells, potentially offering novel therapeutic strategies for the prevention or mitigation of PE.

## Materials and methods

### SFRP2 expression analysis using gene ontology (GO) GSE10588 dataset

Gene expression levels of SFRP2 in PE placental tissue compared with normal pregnancies were analyzed using the GO: GSE10588 dataset, which included data from16 PE placental tissues and 27 normal pregnancies (26). The raw data, acquired as MINiML files, encompassed comprehensive platform, sample and Gene Expression Omnibus records. Following log2 transformation, data normalization was achieved using the preprocessCore package for quantile normalization and limma package for batch effect removal (R; version 3.4.1) (https://cran.r-project.org/). Probe data were mapped to corresponding gene symbols, excluding probes associated with multiple genes. When multiple probes corresponded to a single gene, mean expression value was calculated. Data quality was assessed with boxplots and Principal Component Analysis (PCA) plots showcased samples before and after batch correction. The expression of SFRP2 in PE placental tissue vs. normal pregnancies was analyzed using the Wilcox non-parametric rank test (Wilcox test).

### JEG-3 cell culture

JEG-3 trophoblast cells (cat. no. HTB-36, American Type Culture Collection, ATCC) were cultured in DMEM (cat. no. 10313039) enriched with 10% FBS (cat. no. 16140071) and 1% penicillin/streptomycin (cat. no. 15140122; all Gibco; Thermo Fisher Scientific, Inc.). The cells were incubated in a humidified atmosphere containing 5% CO_2_ at 37°C.

### Vector construction and lentiviral transduction

SFRP2-overexpressing lentiviral vector was engineered utilizing the human SFRP2 genetic sequences obtained from the NCBI GenBank (Gene Bank ID: NM_003013.3) (ncbi.nlm.nih.gov/nuccore/NM_003013.3) by Shanghai GeneChem Co., Ltd. The SFRP2 coding sequence was cloned into GV358 vector (Shanghai GeneChem Co., Ltd.) to generate SFRP2-overexpression (OE-SFRP2) vector. JEG-3 cells were cultured in 6-well plates at 37°C for 36 h and, upon reaching ~70% confluency, transfected using Lipofectamine 2000 (Invitrogen; Thermo Fisher Scientific, Inc.) for 48 h at 37°C. Equal amounts (100 µl) of SFRP2-OE or negative control (NC; empty vector) lentiviral particles were introduced to each well. At 48 h post-transfection, the JEG-3 cells were collected for analysis.

### Reverse transcription-quantitative (RT-q)PCR

Total RNA was extracted from JEG-3 cells using the RNA-iSo PluS kit (cat. no. 9109; Takara Biotechnology Co., Ltd.). RNA was reverse-transcribed using cDNA Synthesis SuperMix (Shanghai Yeasen Biotechnology Co., Ltd.; cat. no. 11119ES60). RT was performed as follows: Initial hold at 25°C for 5 min, followed by 42°C for 30 min and final elongation at 85°C for 5 min. Synthesized cDNA was subjected to qPCR amplification using SYBR Green qPCR Mix (MedChemExpress; cat. no. HY-K0501A) and analyzed on ABI 7500 Real-Time PCR system (Thermo Fisher Scientific, Inc.). Each 20 µl qPCR reaction mixture included 1.0 cDNA, 10.0 SYBR Premix Ex Taq (2X) and 0.4 µl each primer (10 µM) and was completed with double-distilled water. Amplifications followed a two-step cycling protocol as follows: Initial denaturation at 95°C for 30 sec, followed by 40 cycles of 10 sec at 95°C and 30 sec at 60°C. The final step comprised the melting curve analysis. To normalize data, SFRP2 expression was compared with housekeeping gene GAPDH using the 2^−ΔΔCq^ method (27). The specific primer sequences were as follows: GAPDH forward, 5′-CCAGGTGGTCTCCTCTGA-3′ and reverse, 5′-GCTGTAGCCAAATTCGTTG-3′ and SFRP2 forward, 5′-CACCGAGGAAGCTCCAAAG-3′ and reverse, 5′-CTTTCGGACACACCGTTCAG-3′.

### Western blotting

Total proteins were extracted from JEG-3 cells utilizing RIPA lysis buffer (cat. no. HY-K1001; MedChemExpress). Following extraction, the concentration of the extracted proteins was determined using BCA Protein Assay kit (cat. no. 23225; Thermo Fisher Scientific, Inc.). Aliquot containing 5 µg isolated proteins was combined with 5X SDS sample buffer and subjected to electrophoretic separation on 12% SDS-polyacrylamide gel. The separated proteins were transferred onto a PVDF membrane. To minimize non-specific binding, membranes were blocked with 5% non-fat milk at room temperature for 2 h before overnight incubation at 4°C with the following primary antibodies (all 1:1,000): Rabbit anti-SFRP2 (cat. no. ab137560; Abcam), anti-Wnt3a (cat. no. ab219412; Abcam), anti-Axin2 (cat. no. ab109307; Abcam), anti-CyclinD1 (cat. no. ab16663; Abcam), anti-c-Myc (cat. no. ab32072; Abcam), anti-Bax (cat. no. ab32503; Abcam), anti-cleav-caspase 3 (cat. no. ab2302; Abcam), anti-BCL-2 (cat. no. ab32124; Abcam), anti-MMP9 (cat. no. ab228402; Abcam), anti-E-cadherin (cat. no. ab40772; Abcam), anti-β-catenin (cat. no. ab246504; Abcam), anti-p-β-catenin (cat. no. ab314502; Abcam), anti-p-GSK3β (cat. no. ab75814; Abcam), anti-GSK3β (cat. no. ab32391; Abcam) and anti-GAPDH (cat. no. 181602; Abcam). Membranes were washed five times of 10 min each in 0.5% Tris-buffered saline with Tween 20 (TBST) and incubated at room temperature for 2 h with horseradish peroxidase-conjugated secondary goat anti-rabbit antibody (1:10,000; cat. no. ab6721; Abcam). Subsequently, the targeted protein bands were visualized using ECL kit (Thermo Fisher Scientific, Inc.). Quantification of protein levels was performed by densitometric scanning with ImageJ software (ver. 2.0.0, National Institutes of Health, USA).

### Cell viability

OE-SFRP2 JEG-3 cells were seeded in 96-well plates at a density of 1×10^3^ cells/well. Cells were incubated under 37°C with 5% CO_2_. Viability was assessed at 24, 48, 72, 96 and 120 h. At each time point, 10 µl Cell Counting Kit (CCK)-8 assay reagent (cat. no. FC101-03; TransGen Biotech) was added to each well, followed by incubation at 37°C for 3 h. Optical density of each well was measured at 450 nm using the Eppendorf BioPhotometer^®^ D30.

### Cell migration assay

Transwell assay was utilized to assess the migratory capacity of JEG-3 cells post-transfection with OE-SFRP2. Transwell chambers (no. 3414, Corning, Inc.) were applied in the migration assay. Transwell co-culture assay was performed using a 24-well plate. JEG-3 cells were seeded in the upper chamber with 200 µl of serum-free DMEM at a density of 7×10^3^ cells/well. The lower chamber was filled with 800 µl DMEM with 10% FBS. Following 48 h incubation at 37°C, the JEG-3 cells in the basolateral chamber were washed twice with PBS. Subsequently, these cells were stained using 1% crystal violet for 30 min at room temperature. Following a second wash with PBS, stained JEG-3 cells were visualized and photographed using a high-resolution light microscope Olympus cX2 at 100× magnification.

### Flow cytometry

To evaluate apoptosis of JEG-3 cells, Annexin V-FITC and PI staining kit was used (cat. no. A211-01/02; Vazyme Biotech Co., Ltd.) according to the manufacturer's instructions. Cells were analyzed on the Beckman DXI800 (Beckman Instruments, California). Apoptotic cells (PI^+^) were assessed using FlowJo software (version 10.1r5, Tree Star, Inc.). Apoptotic rate (%)=percentage of early + late apoptotic cells.

### EdU staining assay

After incubation in a 24-well plate (1×10^4^ cells/ml) at 37°C for 48 h, JEG-3 cells were treated with EdU (cat. no. A10044, Invitrogen; Thermo Fisher Scientific, Inc.) at room temperature for 2 h. Subsequently, cells underwent 2 min PBS washing, 30 min fixing with 4% paraformaldehyde at room temperature, 10 min permeabilization in 1.0% Triton X-100 and 1 h blocking with a blocking buffer at room temperature. Cells were co-incubated with click reaction solution for 30 min at room temperature in a dark environment. 500 µl of 1× PBS containing 0.5 µl of DAPI solution (no. C1003, Beyotime, China) was used to stain the cell nuclei. The cells were incubated for 30 min at 37°C for staining. Afterwards, they were washed with 1× PBS and then fixed in a solution of 90% glycerol in 1X PBS. EdU visualization was performed using the Click-iT^®^ EdU kit (Invitrogen; Thermo Fisher Scientific, Inc.) and observed under a fluorescence microscope Olympus cX2 at 400×. Image analysis was conducted using Image-Pro Plus v6.0 (Media Cybernetics, Inc.).

### Statistical analysis

Quantitative data generated were analyzed using GraphPad Prism software (Version 8.0; Dotmatics). All experiments were replicated three times to ensure reliability. Data are presented as the mean ± standard deviation. For pairwise group differentiations, unpaired Student's t test was used. For multiple group comparisons, one-way ANOVA was used, followed by Tukey's post hoc test. P<0.05 was considered to indicate a statistically significant difference.

## Results

### SFRP2 is upregulated in PE placental tissue

**Figure 1.**
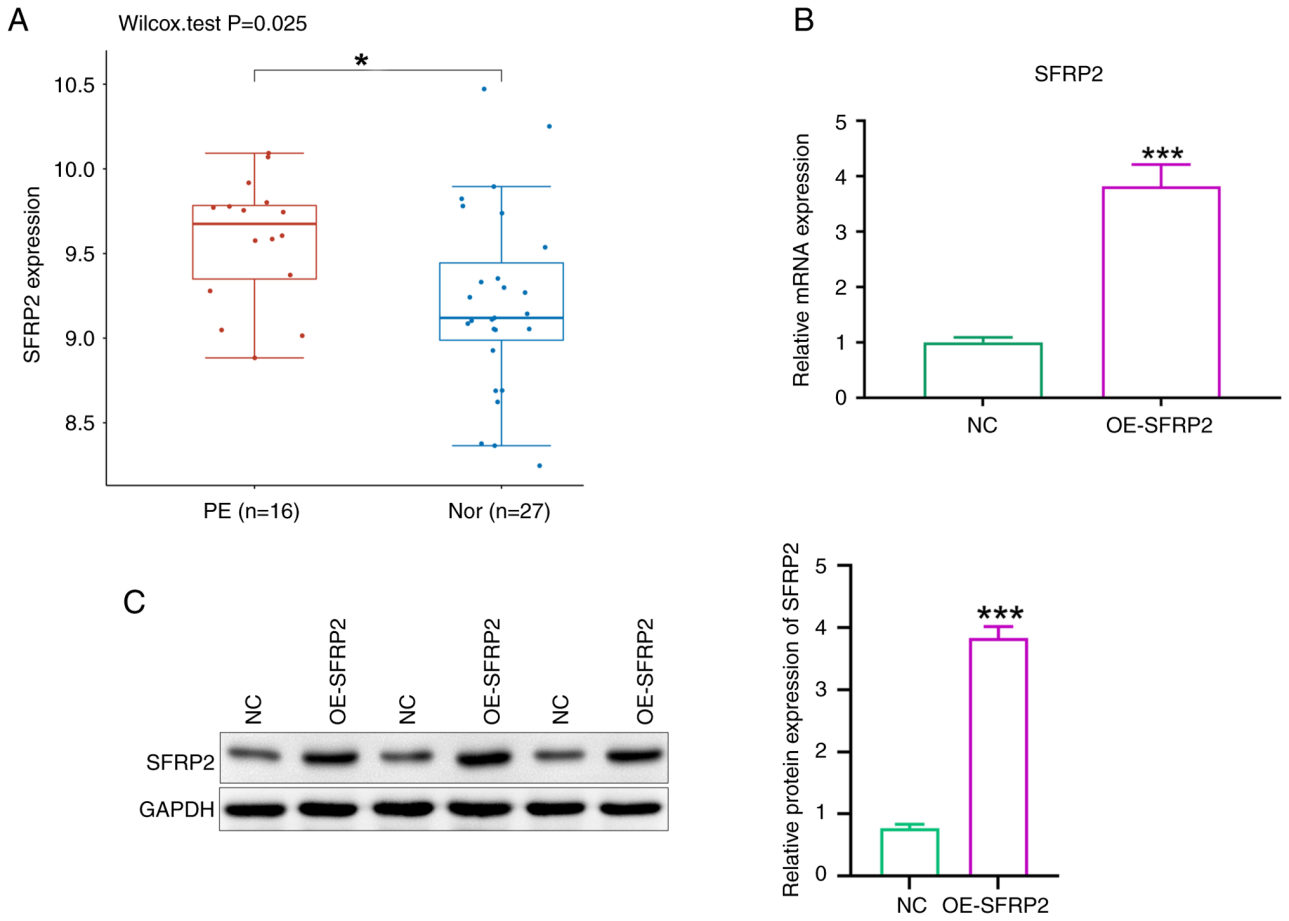
SFRP2 is upregulated in JEG-3 cells. (A) GSE10588 dataset demonstrated upregulated expression of SFRP2 in PE placental tissue compared with that in Nor pregnancy. mRNA and protein expression of SFRP2 in JEG-3 cells was determined by (B) quantitative PCR and (C) western blotting. Grayscale analysis was performed by ImageJ software. *P<0.05, ***P<0.001 vs. NC. PE, preeclampsia; Nor, normal; NC, negative control; OE, overexpression; SFRP2, secreted frizzled-related protein 2.

GO: GSE10588 dataset documents gene expression in placental tissues between patients with PE and those with normal pregnancies. SFRP2 was significantly upregulated in PE placental tissues (Fig. 1A). SFRP2 was overexpressed in JEG-3 trophoblast cells using lentiviral transfection. RT-qPCR and western blot analyses confirmed significant increases in both mRNA and protein levels of SFRP2 in transfected cells compared with controls (Fig. 1B and C).

### Elevated SFRP2 impedes viability and migratory capabilities of trophoblast cells while augmenting apoptosis

**Figure 2.**
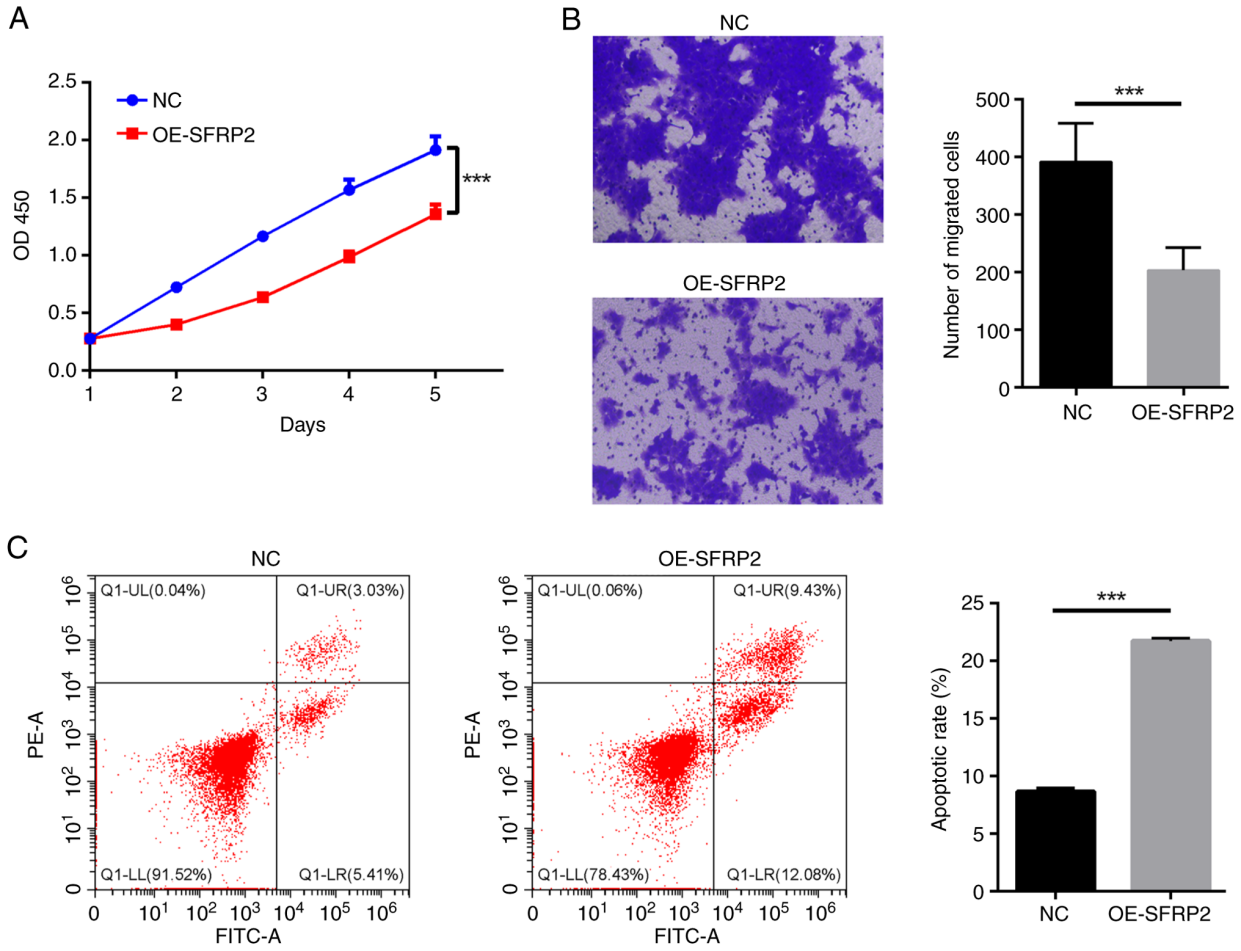
Elevated SFRP2 inhibits viability and migration while increasing apoptosis of trophoblast cells. (A) Viability of JEG-3 cells was detected by Cell Counting Kit-8. (B) Migration of JEG-3 cells was determined by Transwell assay. Magnification, ×100. (C) Ratio of apoptosis of JEG-3 cells on flow cytometric detection. ***P<0.001. NC, negative control; OE, overexpression; SFRP2, secreted frizzled-related protein 2; OD, optical density.

**Figure 3.**
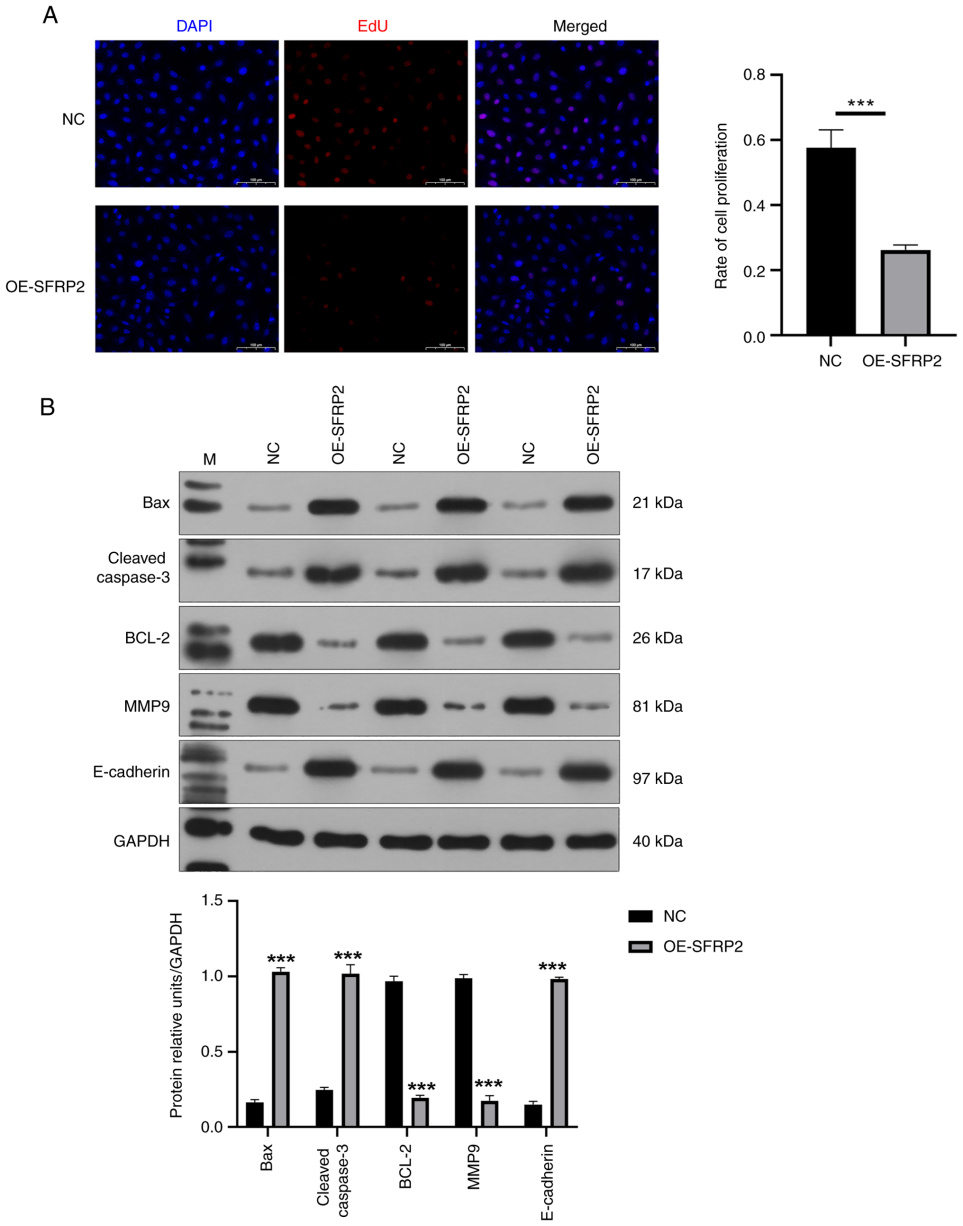
SFRP2 overexpression inhibits migration and promotes apoptosis of trophoblast cells. (A) JEG-3 cell proliferation was determined using EdU staining assay. (B) Protein expression of Bax, cleav-caspase 3, BCL-2, MMP9 and E-cadherin by western blotting. Grayscale analysis was performed by ImageJ software. ***P<0.001. NC, negative control; OE, overexpression; SFRP2, secreted frizzled-related protein 2; cleav, cleaved; M, marker.

Elevated SFRP2 led to a notable reduction in viability of JEG-3 cells demonstrated by CCK-8 assay (Fig. 2A). The results indicated that SFRP2 plays a significant role in regulating cellular processes in JEG-3 cells. In addition, high SFRP2 expression hindered the migration ability of JEG-3 cells, confirmed by Transwell migration assay (Fig. 2B). High SFRP2 expression was concomitant with an elevated apoptosis rate of JEG-3 trophoblast cells, verified by flow cytometric analysis (Fig. 2C). There was a significant reduction in EdU-positive cell number following SFRP2 OE compared with NC (Fig. 3A), indicating that SFRP2 OE exerts an inhibitory effect on cellular proliferation. Furthermore, OE-SFRP2 significantly increased the protein levels of Bax, cleaved-caspase-3 and E-cadherin, while simultaneously reducing levels of BCL-2 and MMP9 (Fig. 3B). These findings strongly indicate that heightened SFRP2 expression impeded proliferative and migratory capacity of JEG-3 cells.

### Augmented expression of SFRP2 inhibits the Wnt signaling cascade

**Figure 4.**
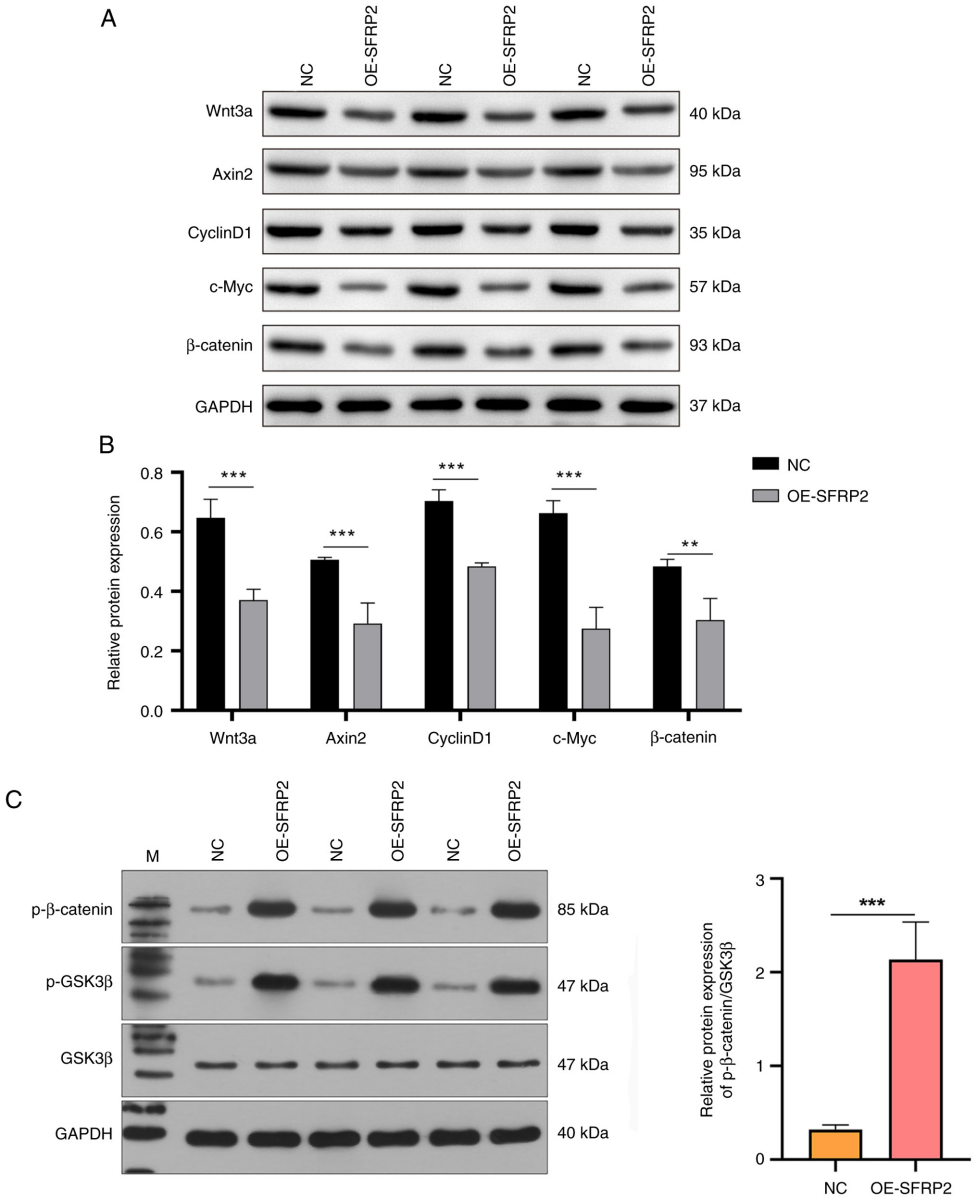
High SFRP2 expression attenuates Wnt signaling. (A) Protein expression of Wnt3a, Axin2, CyclinD1, c-Myc and β-catenin verified by western blot. (B) Grayscale analysis was performed by ImageJ software. (C) Protein levels of p-β-catenin, p-GSK3β and GSK3β in OE-SFRP2 JEG-3 cells verified by western blot. **P<0.05, ***P<0.001. NC, negative control; OE, overexpression; SFRP2, secreted frizzled-related protein 2; p-, phosphorylated; GSK, Glycogen Synthase Kinase 3β; M, protein Marker.

The effect of SFRP2, known for its antagonistic interaction with the Wnt pathway, particularly by competing for Wnt binding to its Frizzled receptor (25), on Wnt signaling components was assessed. Enhanced SFRP2 expression significantly decreased the protein expression of Wnt3a, Axin2, CyclinD1, c-Myc and β-catenin, verified by western blotting (Fig. 4A and B). Furthermore, protein levels of phosphorylated and total β-catenin and GSK3β in JEG3 cells were assessed. OE-SFRP2 expression notably elevated the levels of phosphorylated β-catenin and p-GSK3β, while exerting negligible influence on total GSK3β levels (Fig. 4C). Ratio of phosphorylated β-catenin to GSK3β was notably higher in OE-SFRP2 cells (Fig. 4C), suggesting elevated SFRP2 affected the migration capability of trophoblast cells primarily by modulating the Wnt signaling pathway.

## Discussion

PE is closely linked to impaired invasion capabilities of fetal trophoblast cells, leading to insufficient uteroplacental perfusion (28). The complex regulation of trophoblast cells is key to understanding PE pathophysiology. Here, SFRP2 was upregulated in PE placental tissue. OE-SFRP2 cells significantly decreased viability, proliferation and migration of JEG-3 cells, while promoting apoptosis. These findings align with the hypothesis that SFRP2 serves a pivotal role in PE pathogenesis (19–21). SFRP2 is known to antagonize the Wnt signaling cascade, primarily by competing for Wnt binding to its Frizzled receptor (25). OE-SFRP2 was associated with significant suppression of Wnt-associated gene expression in JEG-3 cells. Consequently, it was hypothesized that elevated SFRP2 levels potentially modulate PE progression by inhibiting the Wnt/β-catenin signaling pathway, leading to decreased migration of JEG-3 cells. This underscores the potential of SFRP2 as a novel molecular target in strategic management of PE.

Wnt signaling pathways, encompassing both the canonical (Wnt/β-catenin) and non-canonical (Wnt/planner cell polarity and Wnt/Ca^2+^pathway) paradigms, serve a central role in cellular activities (29). In adult mammals, Wnts are pivotal in regulating a majority of tissue stem cell types (30). Notably, the inhibition of Wnt5a in activated human hepatic stellate cells markedly hinders their proliferation and leads to the downregulation of type I collagen and TGF-β1 expressions (31). In addition, the WNT/β-catenin pathway plays an essential role in controlling the proliferation of basal layer cells, crucial for maintaining skin homeostasis, especially under pathological conditions (32). Perturbations in the Wnt/β-catenin signaling cascade are associated a range of pathological conditions, including obstetrical and gynecological disorder, metabolic aberration and neoplasms (33). Specifically, Wnt signaling is crucial in trophoblast differentiation and invasion (34–36). Key constituents of the Wnt signaling axis, such as Dickkopf-1 (DKK1) and SFRP2, undergo hypermethylation in trophoblasts. This epigenetic anomaly leads to abnormal activation of the Wnt signaling pathway (37,38). DKK1 can induce apoptosis in JEG3 and BeWo trophoblast cell lines by stimulating the mitochondrial apoptosis pathway (38). Here, OE-SFRP2 significantly inhibited viability and migration of JEG-3 cells, while increasing apoptosis. Bcl-2, Bax and caspase 3 are key proteins involved in the apoptotic process (39). OE-SFRP2 expression increased Bax levels, decreased Bcl-2 levels and activated caspase 3, resulting in apoptosis. Furthermore, MMP9 and E-cadherin, key proteins associated with invasion and metastasis, were inhibited and increased by SFRP2 upregulation, respectively (23,40). OE-SFRP2 decreased invasive capability of JEG-3 cells, as evidenced by downregulation of MMP9 and the upregulation of E-cadherin. These findings confirm the pivotal roles that Wnt inhibitors play in modulating the invasive characteristics of trophoblast cells.

The function of Wnt pathway signaling depends on β-catenin, a key mediator. When β-catenin reaches a key intracytoplasmic concentration, it translocates to the nucleus. There, it interacts with the T cell factor/Lymphoid enhancer-binding factor (TCF/LEF) transcriptional complex, leading to modulation of target genes, including cyclin D and c-Myc. Such transcriptional regulation can lead to anomalies in cellular processes, such as proliferation, invasion and apoptosis (29). There is a significant reduction in β-catenin levels in placental tissues derived from PE (41). This decrease in β-catenin is hypothesized to mediate the inhibitory effects of SFRP5 on the migratory and invasive characteristics of human trophoblast HTR8/SVneo cells (42), emphasizing the key role of the Wnt/β-catenin signaling axis in the pathogenesis of PE. In the present study, OE-SFRP2 lead to a notable decrease in the protein levels of Wnt3a, Axin2, CyclinD1 and c-Myc and the ratio of phosphorylated β-catenin to GSK3β. This suggested that SFRP2 inhibited trophoblast invasion by attenuating the Wnt/β-catenin pathway.

The current therapeutic options for PE are limited, with existing treatments showing restricted effectiveness. Antihypertensive drugs and magnesium sulfate aid in managing preeclampsia-related seizures, but delivery of the fetus and placenta remains the definitive treatment (43). These drugs, however, carry risks of severe systemic toxicities, partly due to their low molecular weights enabling placental transfer and potential fetal harm (44). Preeclampsia poses immediate pregnancy risks and long-term health impacts for both mother and child. Women with preeclampsia have a higher risk of cardiovascular diseases including heart failure, hypertension, and stroke, while infants from preeclamptic pregnancies often have low birth weights, increasing their risk of early adult stroke, heart disease, and metabolic disorders (45,46). Therefore, there is an urgent need to develop targeted therapies for PE. Here, OE-SFRP2 negatively regulated trophoblast viability and migration potential. This effect may be mechanistically linked to disturbances in the Wnt/β-catenin pathway and its downstream effector molecules CyclinD1 and c-Myc. Based on the present results, it was hypothesized that high SFRP2 expression may be closely related to trophoblast dysregulation and the pathogenesis of PE. Targeting SFRP2 may be a viable therapeutic panacea for PE. In conclusion, the present study introduced a new perspective in the etiology and pathogenesis of PE, revealing potential molecular targets that could form the basis of future therapeutic strategies.

The present study highlighted the crucial role of high SFRP2 expression in the etiology of PE, although there are methodological limitations. The *in vitro* experiments were conducted solely utilizing JEG3 cells, a commonly employed choriocarcinoma cell line representative of villous trophoblast cells (47,48). However, use of immortalized human chorionic trophoblast cells, such as HTR-8 cells, is increasingly recognized as a more refined model for studying trophoblast functionality (49,50). Therefore, future research should include HTR-8 cells to validate the present findings. OE-FRP2 markedly attenuated the viability and migratory capacity of JEG-3 cells. Initial tests with JEG-3 cells revealed compromised clonogenic potential and wound healing assay delineated irregular proliferation margins, raising questions about the reliability of these methods (data not shown). Consequently, the lack of wound healing and colony formation assay results constitutes a notable limitation. Additionally, increased SFRP2 expression could potentially impede trophoblast cell migration by inhibiting the Wnt/β-catenin signaling cascade. Nonetheless, the present study was limited to overexpressing SFRP2 in JEG3 cells via lentiviral transfection without exploring the impact of SFRP2 knockdown on JEG3 cell migration and the Wnt pathway. Moreover, the effect of SFRP2 on the Wnt/β-catenin pathway in the absence of specific Wnt inhibitors or activators needs confirmation. Significant upregulation of SFRP2 was observed in PE placental tissues in the GO:GSE10588 dataset. However, lack of clinical PE specimens precluded assessment of SFRP2 expression in PE tissues via immunohistochemistry to elucidate the putative role of SFRP2 in PE pathogenesis. The lack of animal models in this study also curtails the extrapolation of results. Future studies should integrate mouse models to afford a more holistic evaluation of SFRP2 in the pathogenesis of PE.

Increased SFRP2 expression served as a pivotal modulator in PE pathogenesis, predominantly via attenuation of the Wnt/β-catenin signaling cascade, which diminished the migratory attributes of JEG-3 trophoblast cells. Such findings provide potential avenues for innovative therapeutic strategies in the management of PE.

## Data Availability

The data generated in the present study are included in the figures and/or tables of this article.
